# Initial mechanical conditions within an optimized bone scaffold do not ensure bone regeneration – an *in silico* analysis

**DOI:** 10.1007/s10237-021-01472-2

**Published:** 2021-06-07

**Authors:** Camille Perier-Metz, Georg N. Duda, Sara Checa

**Affiliations:** 1 Julius Wolff Institute, Berlin Institute of Health at Charité - Universitätsmedizin Berlin, Berlin, Germany; 2 MINES ParisTech – PSL Research University, Paris, France

**Keywords:** Bone scaffold, Bone regeneration, Scaffold design optimization, Computational mechano-biology

## Abstract

Large bone defects remain a clinical challenge because they do not heal spontaneously. 3-D printed scaffolds are a promising treatment option for such critical defects. Recent scaffold design strategies have made use of computer modelling techniques to optimize scaffold design. In particular, scaffold geometries have been optimized to avoid mechanical failure and recently also to provide a distinct mechanical stimulation to cells within the scaffold pores. This way, mechanical strain levels are optimized to favour the bone tissue formation. However, bone regeneration is a highly dynamic process where the mechanical conditions immediately after surgery might not ensure optimal regeneration throughout healing. Here, we investigated *in silico* whether scaffolds presenting optimal mechanical conditions for bone regeneration immediately after surgery also present an optimal design for the full regeneration process. A computer framework, combining an automatic parametric scaffold design generation with a mechano-biological bone regeneration model, was developed to predict the level of regenerated bone volume for a large range of scaffold designs and to compare it with the scaffold pore volume fraction under favourable mechanical stimuli immediately after surgery. We found that many scaffold designs could be considered as highly beneficial for bone healing immediately after surgery; however, most of them did not show optimal bone formation in later regenerative phases. This study allowed to gain a more thorough understanding of the effect of scaffold geometry changes on bone regeneration and how to maximize regenerated bone volume in the long term.

**Supplementary Information:**

The online version contains supplementary material available at 10.1007/s10237-021-01472-2.

## Introduction

Large bone defects remain a clinical challenge because they do not heal spontaneously. Their gold standard treatment (autologous bone grafting) has several drawbacks such as the need for a second surgery with associated risks and a limited availability of bone graft tissue (Dimitriou et al. 2011; Schlundt et al. 2018). Synthetic scaffolds, e.g. made of metal, polymers or ceramics, appear as a promising treatment alternative for such critical bone defects with several pre-clinical studies reporting successful applications (Reichert et al. 2012; Lovati et al. 2016; Pobloth et al. 2018; Reznikov et al. 2019; Crovace et al. 2020). However, their translation to the clinic remains a challenge in part due to a lack of understanding of the influence of scaffold design on the regeneration process.

Although scaffold design has mainly relied on a trial and error approach so far, recently several research groups have adopted a more systematic approach using numerical or computational optimization methods to maximize or minimize specific properties (e.g. maximum stiffness; maximum permeability for good nutrient flow) (Guest and Prévost 2006; Almeida and da Silva Bártolo 2010; Chen et al. 2011; Xiao et al. 2012; Dias et al. 2014; Wang et al. 2016; Langelaar 2016; Uth et al. 2017; Metz et al. 2020). However, maximizing scaffold mechanical properties might not ensure a better bone regeneration, as too stiff constructs (apparent Young’s modulus greater than 2 GPa) have been shown to achieve lower bone regeneration (Pobloth et al. 2018; Reznikov et al. 2019). Other groups have developed scaffold designs with target values for stiffness and/or diffusivity that would be similar to the tissue being replaced (Hollister et al. 2002; Hollister and Lin 2007; Sturm et al. 2010; Wieding et al. 2014; Makowski and Kuś 2016; Chang et al. 2017). Nonetheless, building a scaffold mimicking the missing tissue mechanical properties cannot ensure to best support endogenous bone regeneration and implant osseointegration (Petersen et al. 2018).

More recently, mechano-biological computer approaches have been used to optimize scaffold design (Boccaccio et al. 2016a). These computer models aim to integrate the existing knowledge on the biology of bone regeneration taking into account experimental observations of the influence of mechanical signals on the bone healing process (Prendergast et al. 1997; Carter et al. 1998; Claes and Heigele 1999). Using this knowledge, Boccaccio and colleagues designed optimized periodic scaffolds so that they would provide favourable mechanical conditions for bone regeneration immediately after implantation (Boccaccio et al. 2016b, a, 2018b, a; Rodríguez-Montaño et al. 2019; Percoco et al. 2020). However, their approach did not account for the whole bone regeneration process but their scaffold design was only optimized for the situation immediately after surgery. Similar studies were conducted for cartilage defect scaffolds (Kelly and Prendergast 2006; Koh et al. 2019). To our knowledge, there were only two time-dependent, mechano-biology-based bone scaffold optimization studies: Poh and colleagues optimized scaffold porosity distribution based on a simplified bone regeneration model (Poh et al. 2019); however, their model allowed only a 1-D optimization. More recently, Wu and colleagues performed a time-dependent mechano-biology-based topology optimization of a large defect scaffold in 2-D and a partial defect scaffold in 3-D (Wu et al. 2021).

Bone regeneration is a highly dynamic process where different types of tissues are formed, remodelled and resorbed in specific locations over the regeneration period, creating a mechanical environment that changes over time. This changing mechanical environment in turn further influences tissue formation. The influence of scaffold design on the regeneration process and whether scaffolds optimized for the post-surgery situation are optimal in terms of the healing outcome remains unknown. In fact, a study conducted on spine fusion devices suggests that optimized design for the situation immediately after surgery would not yield optimal bone growth (Bashkuev et al. 2015). An *in silico* comparison between bone regeneration outcome for scaffolds optimized for the situation immediately after surgery or taking the healing process into account reached the same conclusion in 2-D (Wu et al. 2021).

Here, we propose an *in silico* framework to investigate the influence of the bone regeneration dynamics on the optimum scaffold design. Our aims were (1) to investigate the effect of scaffold design parameters on the predicted regenerated bone volume at the end of the regeneration process and (2) to compare this outcome between scaffolds that would be considered optimal for the post-surgery situation and those that show best regeneration outcome. To achieve this, a computer framework combining an automatic parametric scaffold design generation with a mechano-biological bone regeneration model was developed.

## Material and methods

### Scaffold-tissue geometry and finite element model

**Fig. 1:**
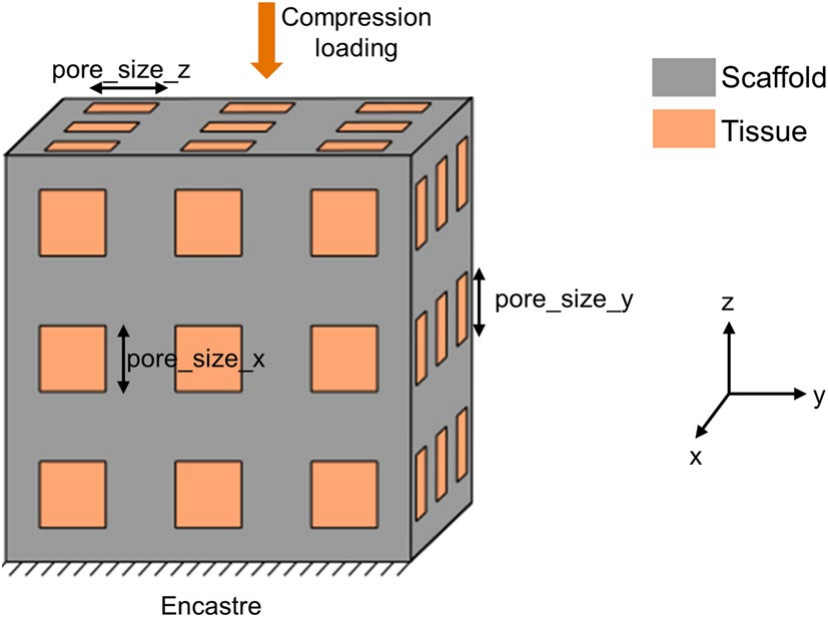
Cubic scaffold representation: scaffold and tissue geometry, parameter definition (pore_size_x, pore_size_y, pore_size_z), loading and boundary conditions

A finite element (FE) model of a cubic scaffold of side 3 mm was designed in Abaqus CAE 2018 (Dassault Systemes Simulia Corp., Rhode Island). 9 square pores were defined by extruded cuts from the different cube faces following each direction (x,y,z); they were positioned following a regular 3*3 grid with 1 mm distance between their centres (Fig. 1). This spacing was in the range of experimentally tested scaffolds (Reichert et al. (2012); Shah et al. (2016)). The pore size could be varied between 0.1 and 0.9 mm, with an identical value for pores along x and y directions (pore_size_x and pore_size_y) and an independent one for pores following the z axis (pore_size_z). The inner scaffold region was modelled as regenerating tissue and was obtained by a Boolean cut operation in Abaqus CAE.

The scaffold was defined as a linear elastic material with Young’s modulus E = 1000 MPa and Poisson ratio $\upnu \,=\,0.3$, within the range of polymer-ceramics composites used in bone regeneration applications (Lam et al. 2008; Reichert et al. 2012). The scaffold pores were assumed to be initially filled with granulation tissue defined as a linear elastic material with Young’s modulus E = 0.2 MPa and Poisson ratio $\upnu \,=\,0.167$ (Checa et al. 2011). Over time, regenerating tissue material properties were updated according to the predicted tissue formation based on a bone regeneration model (see Sect. 2.2).

A tie constraint was defined between regenerating tissue and scaffold. In addition, their lower surfaces were fully constrained both in displacement and rotation (“encastre”). A compression load of 15 N was applied on the top scaffold surface as a distributed load. This loading value was chosen to achieve realistic strain values in the regenerating tissue (0.01-0.1%), comparable to strain ranges determined for scaffolds implanted in long bone defects (Pobloth et al. 2018). Both regenerating tissue and scaffold were meshed with tetrahedral quadratic elements (element type C3D10) of average size 0.1 mm. This size was shown to achieve adequate precision in a preliminary mesh convergence analysis (Online Resource 1).

### Bone regeneration model

A previously described mechano-biological bone healing model validated against fracture healing experimental data (Checa and Prendergast 2009) was used to predict tissue formation within the scaffold pores from 0 to 60 days after implantation. In short, it consisted in a 3-D agent-based computer model implemented in C++. The spacing between agents was defined as $20\,\upmu \hbox {m}$ to account for the average cell size (Isaksson et al. 2008). Each agent represented one cell of one of the following phenotypes: progenitor cell, fibroblast, chondrocyte or osteoblast. Each cell was assumed to deposit its corresponding tissue in the same agent position: granulation tissue, fibrous tissue, cartilage and bone, respectively. Proliferation, apoptosis and differentiation were regulated by a mechanical stimulus based on hydrostatic stress and minimal principal strain defined by Claes and Heigele (Claes and Heigele 1999), with a bone resorption zone (Postigo et al. 2014): in each FE, the local stress and strain measures defined a stimulus that would favour the differentiation and proliferation of exactly one phenotype and the apoptosis of all other phenotypes. Progenitor cells were allowed to migrate randomly with an average speed of $30\,\upmu \hbox {m/h}$ (Appeddu and Shur 1994). They were initially seeded on the top and bottom surfaces (30% occupancy) of the regenerating tissue, similar to bone marrow cell sources in a large bone defect (Checa and Prendergast 2009). The rest of the tissue volume was considered cell-free at the initial time point.

This agent-based model was coupled with the FE model in two ways: (1) the mechanical stimulus, derived element-wise from the FE analysis, influenced cell behaviour; and (2) the tissue material properties were updated in the FE model at every iteration depending on the tissue distribution. More precisely, each element of the FE model was mapped to the agents it contained and its material properties were defined as a weighted average of the tissues predicted in these agents according to a rule of mixtures; these quantities were further averaged over the last ten iterations to account for tissue deposition and maturation (Lacroix and Prendergast 2002). The FE analysis and agent-based simulations were run iteratively to predict the full regeneration process, where one iteration represented one day.

### Parametric study set-up

A parametric study of the effect of scaffold geometry on bone regeneration was performed: pore_size_x (=pore_size_y) and pore_size_z were uniformly sampled between 0.1 and 0.9 mm with a spacing of 0.05 mm and a porosity greater than 50%. The porosity of the scaffold was defined as the void fraction divided by the total volume of the cube. Out of the 289 possible scaffold configurations, 181 had a porosity greater than 50% and were therefore simulated. The outcome was defined as:

1. The regenerated bone volume fraction after 60 days according to the bone regeneration model described in Sect. 2.2 - the time of 60 days was chosen as it allowed to achieve an approximately stationary state for the tissue distribution in a scaffold with intermediate-size pores; the fraction was computed as the predicted regenerated bone volume divided by the tissue (scaffold pores) volume;
2. The volume fraction of tissue under bone-favouring initial mechanical signals right after implantation, according to the mechanoregulation theory described in Sect. 2.2; the fraction was computed as the predicted volume under bone-favouring initial mechanical signals divided by the tissue (scaffold pores) volume.

**Fig. 2:**
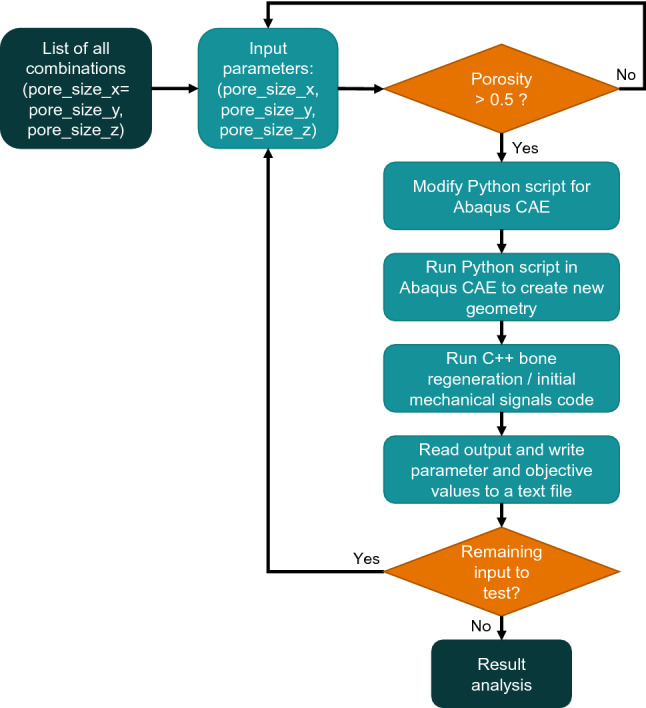
Flow chart of the computational framework developed to investigate the influence of scaffold design on the regeneration process for a large number of scaffold designs

To automate the creation of the 181 scaffold and regenerating tissue FE models, a Python script in Abaqus was developed which allowed to define the pore corner positions based on the pore size. MATLAB R2018b (The MathWorks Inc., Massachusetts) was then used to launch the set of simulations (i.e. bone regeneration model for each scaffold design) and save the corresponding output: (1) regenerated bone volume fraction after 60 days and (2) volume fraction of regenerating tissue under bone-favouring initial mechanical signals. The framework consisted in updating the Python script to create a new FE model, launching the C++ code for bone regeneration and initial mechanical signals evaluation, and reading the output files (Fig. 2). Pore size values (input) and corresponding output were written to a text file for further analysis.

## Results

### Bone regeneration predictions

**Fig. 3:**
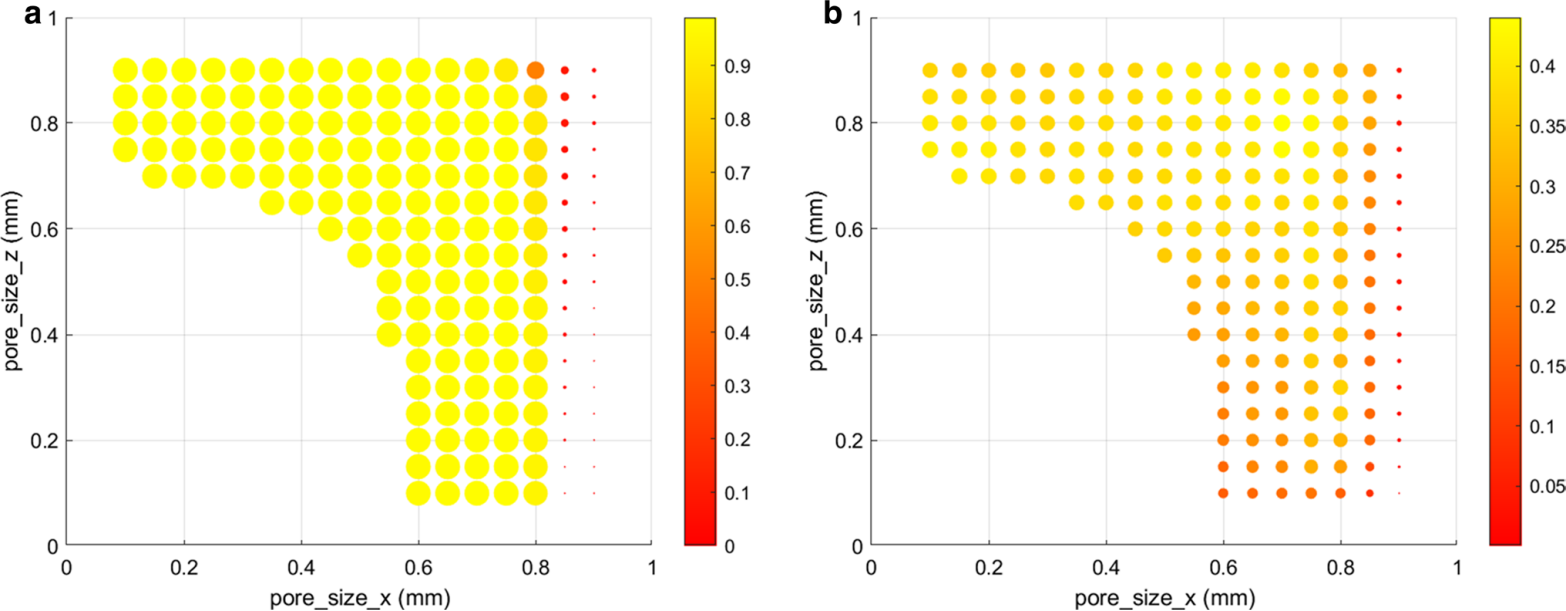
**a** Volume fraction of tissue under bone-favouring initial mechanical signals as a function of pore_size_x and pore_size_z. **b** Predicted regenerated bone volume fraction after 60 days as a function of pore_size_x and pore_size_z. The point size is proportional to the outcome (between 0 and 1)

**Fig. 4:**
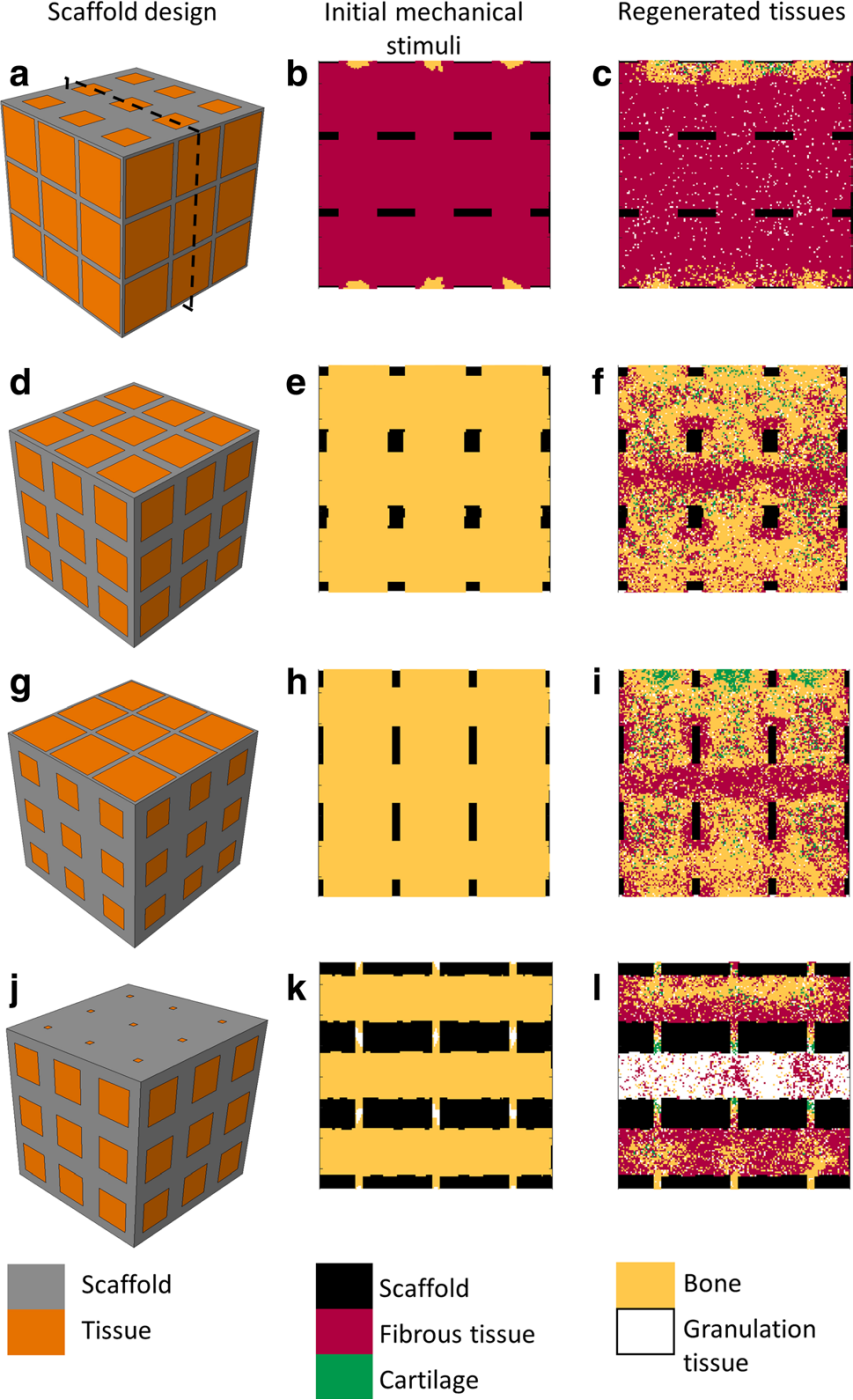
**a**,**b**,**c** Scaffold defined by pore_size_x = 0.9 mm, pore_size_z = 0.5 mm: (**a**) scaffold design, (**b**) tissue types favoured by the initial mechanical signals and (**c**) predicted tissue distribution after 60 days. **d**,**e**,**f** Best scaffold design for optimal bone regeneration (pore_size_x = 0.7 mm, pore_size_z = 0.8 mm). **g**,**h**,**i** Best scaffold design according to the post-surgery initial mechanical signals (pore_size_x = 0.5 mm, pore_size_z = 0.9 mm). **j**,**k**,**l** Scaffold defined by pore_size_x = 0.6 mm, pore_size_z = 0.1 mm). Colour codes for the scaffold design and the favoured or regenerated tissues are given below. The sections with the initial mechanical stimuli and the regenerated tissues are defined as shown with the dotted lines in (**a**)

The 181 scaffold geometries included in the study yielded notably different predicted regenerated bone volume fractions after 60 days, ranging from 5 to 44% of the scaffold pore volume (Fig. 3b). The highest regenerated bone volume fraction was predicted with pore_size_x = 0.7 mm and pore_size_z = 0.8 mm. The corresponding scaffold geometry and final tissue distribution after 60 days are depicted in Fig. 4d, f, respectively: bone was predicted within the scaffold pores; however, in the centre region and surrounding the scaffold walls, an area of fibrous tissue formation was observed.

Very high pore_size_x dimensions - corresponding to large pores in the horizontal directions - consistently resulted in bad-performing designs for bone regeneration: they yielded very high porosities and much deformation, mostly leading to fibrocartilage formation (Fig. 4a, c). Scaffolds with low pore dimension in the vertical direction, pore_size_z, also led to poor healing outcomes. Interestingly, close to optimal bone regeneration outcome was also found for scaffolds with high pore_size_z and low pore_size_x (Fig. 3b).

### Initial mechanical signal predictions

The 181 scaffold geometries included in the study yielded even more marked differences when looking at the initial mechanical signals within the scaffold pores: the tissue volume fraction under bone-favouring initial mechanical signals varied from 5 to 99% (Fig. 3a). However, there were many designs close to the optimum value: 102 different designs showed more than 98% bone-favouring initial signals (large yellow points on Fig. 3a).

**Fig. 5:**
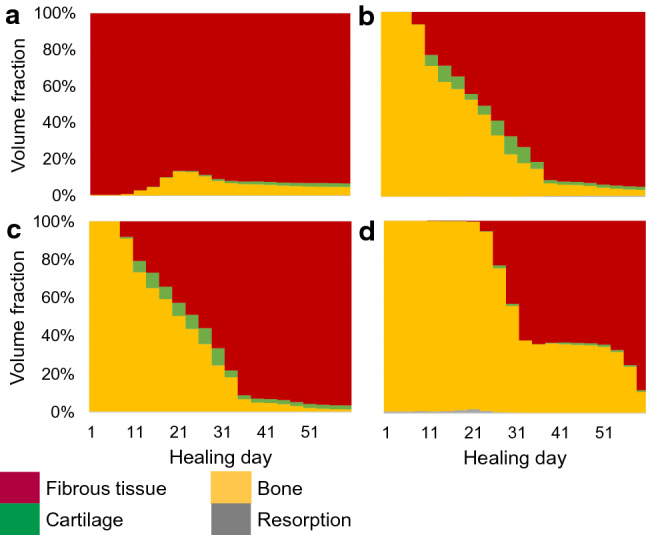
Time evolution of the tissue volume fractions under each mechanoregulation stimulus (resorption, bone, cartilage, fibrous tissue) for: **a** the scaffold defined by pore_size_x = 0.9 mm, pore_size_z = 0.5 mm; **b** the scaffold defined by pore_size_x = 0.7 mm, pore_size_z = 0.8 mm; **c** the scaffold defined by pore_size_x = 0.5 mm, pore_size_z = 0.9 mm; **d** the scaffold defined by pore_size_x = 0.6 mm, pore_size_z = 0.1 mm. Note: Previously deposited bone is not resorbed in presence of higher stimuli (e.g. favouring cartilage or fibrous tissue), what explains the discrepancy between the tissue volume fraction under bone-favouring mechanical signals at the end of the healing process and the actual bone volume fraction

Highest bone-favouring volume fraction was predicted with pore_size_x = 0.5 mm and pore_size_z = 0.9 mm. The corresponding scaffold geometry and final tissue distribution after 60 days are depicted in Fig. 4g, i. More fibrous tissue and cartilage were predicted compared to the best design obtained according to the bone regeneration volume (Fig. 4f): bone occupied 40% of the tissue volume instead of 44%. Moreover, the initial mechanical signals favoured 99% of the volume to become bone, far from the prediction after healing time (Fig. 4h, i). A similar discrepancy between initial and final prediction could be seen for the actual best design: 99% favourable initial signals instead of 44% predicted regenerated bone (Fig. 4e, f). In fact, the mechanical signals showed a dynamic evolution over healing time due to the gradual tissue deposition (Fig. 5); in particular, the hydrostatic stress increase over time led to mechanical signals favouring more and more fibrous tissue.

Also here, very large pores in the horizontal directions resulted in less favourable designs. Already at the initial time-point, those high-porosity designs yielded too much deformation and fibrocartilage-favouring mechanical signals. In addition, we could see no relation between initial and final bone-favouring scaffold designs (comparing Figs 3a and b). For instance, the scaffold defined by pore_size_x = 0.6 mm and pore_size_z = 0.1 mm would be seen as a very good design according to the initial mechanical signals (98% bone-favouring signals), whereas our computer model predicted only 16% regenerated bone after 60 days (Fig. 4j-l). In this case, not only were the mechanical signals after 60 days favouring more fibrocartilage, but also the small vertical pores of size 0.1 mm considerably reduced cell infiltration inside the scaffold, delaying the healing process.

## Discussion

To the authors’ knowledge, this is the first *in silico* study that analyses the time dependency of mechano-biologically optimized scaffolds and compares predicted bone healing outcome with initial mechanical stimuli within 3-D scaffolds across a wide set of scaffold geometries. Based on a computer model for bone regeneration, our results show that optimized initial mechanical conditions within a bone scaffold would not ensure optimal predicted bone regeneration. Indeed, some scaffold designs were predicted to perform remarkably well right after implantation but yielded low bone growth after 60 days. Thus, mechanical properties computed right after scaffold implantation cannot be used as a proxy for bone regeneration success and optimizing the tissue-scaffold mechanical environment in the post-surgery situation will not help fostering bone regeneration.

Only few studies so far conducted scaffold design optimization based on mechano-biological cues. Boccaccio and colleagues performed scaffold parametric optimizations based on the initial mechanical signals and did not take into account the full regeneration process (Boccaccio et al. 2016b, a, 2018b, a; Rodríguez-Montaño et al. 2019; Percoco et al. 2020). The study described in (Boccaccio et al. 2016a) also considered a cubic scaffold configuration with square pores (same dimensions in all space directions) and Young’s modulus 1000 MPa. When subjected to a pressure of 1-2 MPa, their optimal design showed a much lower porosity than in our study and yielded only 30% bone regeneration. However, it should be noted that the cube was smaller (side of 1.913 mm).

Only one study used a mechano-biological algorithm to perform a time-dependent bone scaffold topology optimization in 2-D (large defect) and 3-D (partial defect) (Wu et al. 2021). Based on their bone remodelling computer model, they demonstrated that a superior design for bone regeneration was obtained when optimizing for the full regeneration process compared to the situation immediately after surgery, similar to our results. However, they did not take into account the actual cell invasion of the scaffold pores nor the different tissue types (e.g. fibrous tissue, cartilage). Our results are further in line with a study conducted on spine fusion devices (Bashkuev et al. 2015) that showed that the material properties of a spinal cage optimized to achieve the best mechanical signals within the fusion region immediately after surgery would not be optimal in terms of fusion outcome.

Another parametric study was previously conducted on a cubic bone scaffold to determine the best combination of Young’s modulus, scaffold porosity and dissolution rate to maximize bone formation (Byrne et al. 2007). For a non-degradable scaffold of side 1.913 mm, Young’s modulus 1000 MPa, 1 MPa pressure load and 70% porosity – the case most comparable to our best-case scenario – Byrne and colleagues predicted 43% of regenerated bone after 60 days, very close to our predictions of 44%. In general, they found higher porosities (70%) to be more beneficial for bone in-growth, what is in agreement with our study where the best design porosities ranged from 80 to 87%. However, our study also revealed that the highest porosities ($>90\%$) consistently resulted in impaired healing: they yielded too much deformation, both at the initial time point and during the regeneration process, thus favouring fibrocartilage formation. Apart from that, we found no correlation between porosity and healing outcome, what emphasises the significance of the material distribution over the amount of material, even in such a simple architecture. Notably, our results revealed that the vertical pores (direction of the progenitor cell sources) need to be large enough to ensure a quick invasion of the scaffold and a fast healing. When pores were smaller than ca. 0.4 mm, they yielded low regenerated bone volume after 60 days (cf. e.g. Fig. 4l). On the contrary, the horizontal pores should not be too large ($<0.85\,mm$) to avoid too high deformations. Our results are in agreement with experimental data: pores bigger than ca. $300\,\upmu \hbox {m}$ were shown to achieve good bone regeneration outcome (Zadpoor 2015; Abbasi et al. 2020; Băbţan et al. 2020), and ideal porosities ranged from approximately 70 to 90% in various studies (Shah et al. 2016; Băbţan et al. 2020; Zheng et al. 2020).

To the authors’ knowledge, no mechano-biologically optimized scaffold geometry has been tested in an *in vivo* setting so far. Only a few studies conducted *in vitro* experimental studies with an optimized scaffold design to test the predicted mechanical properties (Challis et al. 2010; Dias et al. 2014; El-Sayed et al. 2020). Further *in vivo* studies investigated specific scaffold design properties such as strut size (Pobloth et al. 2018; De Wild et al. 2018), strut arrangement (Berner et al. 2014) and pore size (Li et al. 2016). Most studies have related the healing outcome with overall scaffold elastic modulus or tissue strains *a posteriori*, suggesting softer (apparent Young’s modulus lower than 1 GPa) and more porous scaffolds to be more beneficial for bone regeneration (Pobloth et al. 2018; De Wild et al. 2018); these conclusions are in agreement with our model predictions, where rather high scaffold porosities correlated with good bone regeneration potential.

This study had several limitations: first and most importantly, we used a fracture healing computer model that has not been validated for scaffold-supported bone regeneration. In fact, in a recent study, we showed that surface guidance is required to explain experimental patterns of bone tissue formation within a titanium scaffold inserted in a large bone defect in sheep (Perier-Metz et al. 2020). Since previous studies on mechano-biological optimization of scaffolds have not taken into account surface guidance effects yet, we decided to omit this effect to be able to compare our results. For clinical applications however, scaffolds should be optimized considering surface guidance effects. Nevertheless, we believe that our conclusions regarding optimization towards initial mechanical signals or the full regeneration process remain. Second, our framework was applied to a very simplified geometry for computational efficiency reasons; more realistic bone scaffold geometries should be tested, e.g. with a cylindrical shape and with bone marrow and cortical bone sub-regions. The loading scenario was also simplified (compression), but bending or torsion should be present as well. In addition, the mechanical load was distributed directly on the top surface which created some high strain regions in the soft tissues at that boundary. However, the effect was very small since we predicted bone formation in this region for most scaffold configurations. The cell distribution at the beginning of the healing process was chosen to replicate a bone defect healing, but should be refined depending on a specific defect geometry and known cell sources from e.g. the periosteum or the bone marrow. Cellular processes were described in a relatively simplistic manner that has been shown to have good prediction capabilities in fracture healing *in silico-in vivo* studies (Prendergast et al. 1997; Claes and Heigele 1999; Lacroix and Prendergast 2002; Isaksson et al. 2008; Khayyeri et al. 2009; Checa et al. 2011; Borgiani et al. 2019). However, their potential to predict bone regeneration within scaffolds should be further evaluated. Other time-dependent properties of real biological tissues could be included to better reflect tissue deposition and maturation dynamics; finer mechanical models could be employed to reproduce anisotropic or non-linear behaviours of the studied tissues. Lastly, to fully investigate the dynamic interaction between scaffold and bone regeneration, scaffold material degradation should be included in future studies; this would add further dynamic mechanical property changes interacting with the regeneration process. However, polymer-composite scaffold material has been observed to degrade at longer time scales (months or years) than the first healing phase studied here (a few weeks) (Lam et al. 2009; Kang et al. 2020). In designs with a very high porosity, scaffold degradation might already play a role early in the healing process; this remains to be investigated.

In summary, we elucidated the effect of scaffold pore sizes on the initial mechanical signals within the scaffold pores and long-term bone regeneration predictions as a parametric study across different scaffold geometries. Not only did our *in silico* study confirm that pore size has a notable effect on bone healing predictions, but more importantly it highlighted the very relevant difference between immediate post-implantation conditions compared to a regeneration process taking 60 days. We propose a technological platform that allows to optimize bone scaffold designs not only against mechanical failure and initial ingrowth of bone but for a long-term optimized dynamic regeneration process. This study points out pore size as a key parameter for such optimization and allows to gain a more thorough understanding on the effect of scaffold geometry changes on bone regeneration. Future work should use similar set-ups to perform scaffold design optimization that also includes scaffold resorption to gain a comprehensive understanding of the various cascades of formation, remodelling and resorption and how taking them into account allows to maximize regenerated bone volume in the long term.

## Supplementary Information

Below is the link to the electronic supplementary material.

Supplementary file1 (PDF 454 kb)
