# Supplementary material for: Initial mechanical conditions within an optimized bone scaffold do not ensure bone regeneration – an in silico analysis
Source: Biomech Model Mechanobiol. 2021 Jun 7;20(5):1723–31. doi: 10.1007/s10237-021-01472-2 (PMC8450217; doi:10.1007/s10237-021-01472-2)
Supplement: Supplementary file 1 — Supplementary file1 (PDF 454 kb) [file 10237_2021_1472_MOESM1_ESM.pdf]

# Online Resource 1 – Mesh convergence analysis

| Refer-ence | Average mesh size | Number of elements (callus) | Number of elements (scaffold) | Run time (s) |
|---|---|---|---|---|
| a | 1 mm | 3940 | 2271 | 19 |
| b | 0.2 mm | 10119 | 4455 | 22 |
| c | 0.1 mm | 37356 | 12536 | 81 |
| d | 0.06 mm | 114114 | 29829 | 1627 |
| e | 0.03 mm | 592430 | 174994 | 32373 |

Table 1: **Mesh convergence analysis parameters** (scaffold defined by pore_size_x = 0.7 mm, pore_size_z = 0.8 mm)

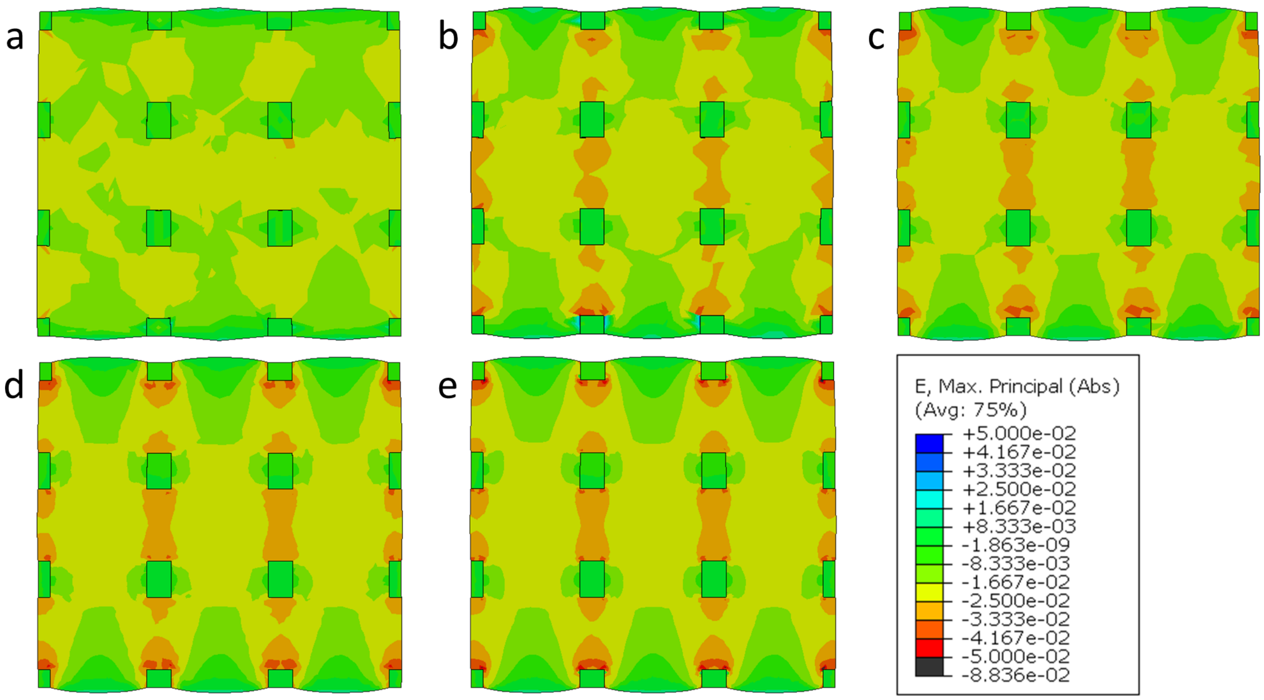


Figure 1: Absolute maximal principal strain distribution in the mid-xz section of the scaffold-tissue construct for average mesh size: (a) 1 mm, (b) 0.2 mm, (c) 0.1 mm, (d) 0.06 mm and (e) 0.03 mm. Strain patterns and values are very similar for all cases except case (a) with 1-mm FE (same order of magnitude than the scaffold pores). In the remaining of the study, 0.1-mm FE were used.
